# Evaluation of a Minigrant Program for Multilevel Interventions to Promote Human Papillomavirus Vaccination in Rural Communities in Georgia

**DOI:** 10.5888/pcd23.250328

**Published:** 2026-08-06

**Authors:** Cam Escoffery, Courtney Petagna, Rithika Chari, Erica Hsu, Rohini Vijay Kadoo, Regine Haardörfer, Yue Guan, Alexandra B. Morshed, Jessica Wells, Sarah Blake, Remy Hutchins

**Affiliations:** 1 Department of Behavioral Sciences and Health Education, Emory University Rollins School of Public Health, Atlanta, Georgia; 2 Nell Hodgson Woodruff School of Nursing, Emory University, Atlanta, Georgia; 3 Department of Health Policy and Management, Emory University Rollins School of Public Health, Atlanta, Georgia; 4 Southwest Health District, 8-2, Division of Public Health, Georgia Department of Public Health, Albany, Georgia

## Abstract

**Introduction:**

Rates of human papillomavirus (HPV) vaccine are lower in rural counties than in urban areas. The objective of the minigrants evaluation was to assess HPV vaccination uptake, factors related to implementation success, and barriers to implementation.

**Methods:**

We conducted a concurrent, mixed-methods evaluation guided by the RE-AIM (Reach, Effectiveness, Adoption, Implementation, and Maintenance) and CFIR (Consolidated Framework for Implementation Science) frameworks. Four health departments received funding, a toolkit with implementation strategies, and monthly technical assistance calls or a learning collaborative for 1 year. The data sources were program documents, staff interviews and surveys, immunization databases, and caregiver or young adult surveys. Key evaluation metrics were 1) reach of patients and adoption of intervention levels (patient, provider, or practice); 2) HPV vaccination effectiveness; 3) implementation perspectives from staff, caregivers, and young adults; 4) implementation barriers and facilitators; and 5) program sustainability capacity.

**Results:**

Most sites adopted 2 of 3 levels (patient, provider, or practice). Common multilevel strategies for series completion were patient education, incentives, provider training, and patient reminders. The total HPV vaccination rate increased 26.7% from 2023 to first quarter 2024, and initiation rate increase was 51.8% across the county departments. Overall, on a scale from 1 to 5, with 1 being “strongly disagree” to 5 being “strongly agree,” sites reported positive experiences with program implementation and perceived the program was feasible (mean, 4.4 points), acceptable (mean, 4.4 points), and appropriate (mean, 4.2 points). Staff members also reported high ease of implementation (mean, 4.0 points) and commitment to program delivery (mean, 4.8 points). The parents or caregivers (n = 20) reported in surveys that they saw educational materials (100%), received educational materials (90%), and received a vaccine reminder (75%). CFIR-related facilitators were priority of the health issues, leadership involvement, technology infrastructure, communications about the vaccine and program, available resources, and staff training at the inner setting and external support at the outer setting. Barriers included limited resources, communication and structural characteristics (eg, technology, issues related to electronic health records), and local attitudes and conditions. The mean program sustainability capacity score was 4.2, with highest scores for evaluation, effectiveness, and workflow integration.

**Conclusion:**

Multilevel intervention strategies can successfully increase HPV vaccination rates in rural settings with implementation supports to address cancer prevalence in rural areas.

**Summary**

**What is already known on this topic?**

The overall human papillomavirus (HPV) vaccination rate among adolescents is suboptimal, and disparities exist in rates of vaccination between metropolitan and rural regions. The effect of multilevel interventions to promote HPV vaccination in rural regions is underreported in the literature.

**What is added by this report?**

This mixed-methods implementation evaluation found that minigrants for implementing multilevel interventions to promote HPV vaccination were effective in increasing HPV vaccine uptake and initiation.

**What are the implications for public health practice?**

Public health agencies and clinics can use multilevel evidence-based strategies to increase HPV vaccination to reduce cancer mortality rates and regional disparities.

## Introduction

Human papillomavirus (HPV) is the most common sexually transmitted infection in the US (1), and nearly 42 million people are currently infected (2). HPV is transmitted through genital contact during vaginal and anal sex, but it can also be passed on during oral sex (2). Low-risk HPV types cause genital warts whereas high-risk HPV types are associated with the following cancers: cervical, vaginal, vulval, penis, anal, and oropharyngeal (3,4). It is estimated that 42,700 cases of HPV-associated cancers will occur in the US each year, including about 24,400 among women and 18,300 among men (3). There are sex differences in the distribution of HPV-related cancers; for example, the prevalence of oropharyngeal cancer is higher among men than women and accounts for 78% of all HPV cancers in men (5). Prevention methods for HPV are primarily focused on adolescents and young adults, as they are most often affected by the virus.

In 2009, the US Food and Drug Administration approved the quadrivalent HPV vaccine for girls and women aged 9 through 26 years to prevent genital warts and anal cancer (6). In late 2016, the Advisory Committee on Immunization Practices recommended a 2-dose schedule for adolescents, including male adolescents, initiating between 9 to 14 years (7). In 2023, data from the National Immunization Survey–Teen found that only 61.4% of adolescents aged 13 to 17 years were up to date with both doses of the HPV vaccine, with female adolescents reporting higher completion rates than male adolescents (64% vs 59%) (8). However, people who were not vaccinated or did not complete the HPV series were male, non-Hispanic or Latino, and living in the US South (9). HPV vaccination rates are lower in Georgia than the national HPV vaccination rates; in 2023, only 40.5% of adolescents aged 13 to 17 years overall were up to date with their vaccinations, and the state was ranked 49th nationally (10). Rural communities also have vaccination rates that are 10% to 15% lower than those in metropolitan areas (11,12). Therefore, HPV vaccination rates are suboptimal both nationally and in Georgia, compared with the Healthy People 2030 goal of 80% (13).

Literature reviews of interventions regarding promotion of the HPV vaccine have been conducted in an effort to increase vaccination rates (14–17). Most studies of the promotion of the HPV vaccine among adolescents and teenagers have examined only individual-level strategies, such as creating informational flyers and media campaigns. However, organizational strategies (eg, school-based vaccination, systems change) to increase HPV vaccination rates have been overlooked and should be considered for broader reach and impact. National initiatives and researchers have been recommending interventions at multiple levels to address different individual-, provider-, community-, and systems-level barriers to prevention (18,19). A recent systematic review found that most interventions to increase HPV vaccination were at a single level, mostly at the parent, adolescent, or young-adult level (20). Research has found that common predictors to vaccine uptake are educating the public through awareness campaigns, provider recommendations, patient reminders, training of providers and teachers (vaccine delivery personnel), and multicomponent interventions (21–23). The *Community Guide* from the Community Preventive Services Task Force recommends using a combination of health care system–based interventions and individual-level approaches to increase vaccination rates in relevant populations (23). Walling et al found that interventions focused on both the parent and provider levels were more successful in increasing HPV vaccine initiation and completion rates (15). Therefore, multilevel interventions should be developed and evaluated to promote HPV vaccine uptake.

Minigrants have been used for health promotion and cancer and chronic disease prevention by giving local organizations funds to support delivery of evidence-based prevention strategies or interventions (24) and are effective in changing cancer-prevention behaviors (25,26). The purpose of this evaluation was to assess the multilevel pilot minigrants program on HPV vaccination rates in rural southwest Georgia and to assess the facilitators and barriers to program delivery.

## Methods

### Study design

This minigrants evaluation was a concurrent mixed-methods study focused on primary and secondary outcomes (27). The primary measures were HPV vaccine initiation and completion rates collected from electronic health records and Georgia’s immunization registry. The secondary measures were program delivery, observations of implementation strategies, and Weiner’s implementation outcomes of acceptability, feasibility, and appropriateness (28). This evaluation was reviewed and deemed exempt by Emory University’s institutional review board.

### Study setting

The purpose of the HPV Vaccine Mini-grants Program was for grantees to implement a multilevel intervention to increase capacity and HPV vaccination rates in rural communities. For the multilevel intervention, grantees were required to focus on at least 2 of the 3 levels (patient, provider, or practice level) (Figure). Multilevel was defined as the use of a combination of more than 1 level of influence on implementation of the intervention including individual, interpersonal, organizational, or community. To be eligible for the minigrants program, grantees had to be a health department, clinic, health system, or student health center located in southwest Georgia with the ability to offer HPV vaccination. Emory Cancer Prevention and Control Research Network (CPCRN) hosted a 1-hour informational webinar over Zoom where interested organizations attended to find out more about the minigrants program. Organizations that were interested after attending the webinar completed a minigrants application, and a review committee selected 4 final grantees by using a standardized scoring rubric. All 4 grantees were county health departments in southwest Georgia. The program took place from January to December 2023. During this time, Emory CPCRN provided up to $10,000, a toolkit with evidence-based strategies for each of the 3 levels, and technical assistance calls each month. During the monthly calls, the grantees shared their progress and received technical assistance either individually or with all 4 grantees as a group. They also submitted completed activities in workplans.

**Figure:**
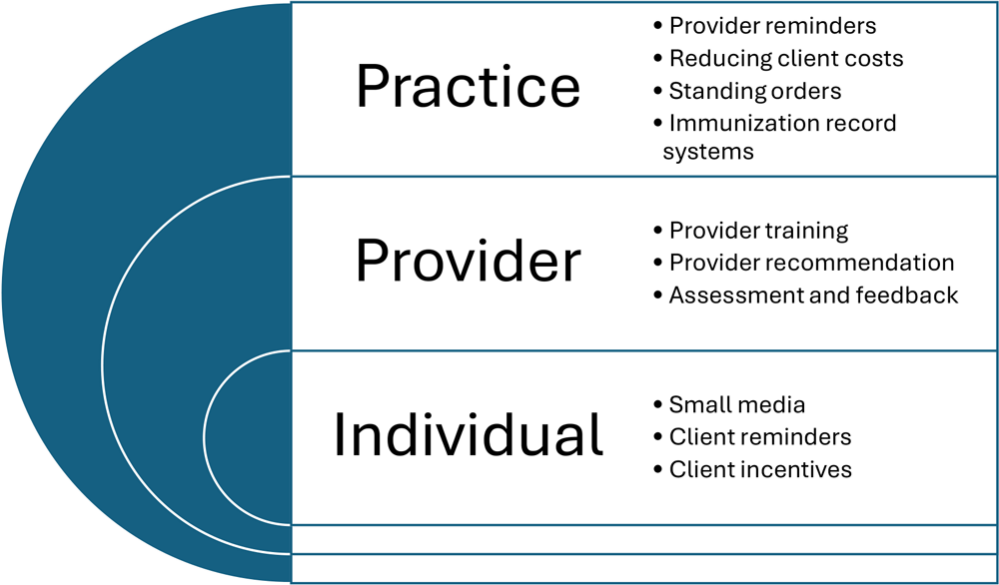
Components of a multilevel minigrant intervention to promote HPV vaccination among young adults in rural Georgia, 2023–2024.

### Study data and measures

The overall theoretical frameworks guiding the evaluation were RE-AIM (Reach, Effectiveness, Adoption, Implementation, and Maintenance) (29) and the Consolidated Framework for Implementation Science (CFIR) (30). RE-AIM framed the overall implementation evaluation in terms of indicators of adoption and reach, description of implementation of the multilevel strategies, HPV outcomes, and comments about maintenance of the minigrants program. CFIR domains were selected to describe factors that assisted with implementation. This mixed evaluation study included caregiver, patient, and staff surveys, and staff interviews during and after implementation in spring 2024. All participants received a $25 gift card when completing a survey or interview. A staff member could receive up to 2 gift cards if they completed both the survey and interview.

*HPV vaccine outcomes*. The primary outcome measure of HPV vaccines came from the health department electronic medical records. We collected information on total doses and initiation rates from the district health office.

*Intervention surveys*. Data were collected via 3 separate surveys: 1) caregiver, 2) young adult, and 3) staff. The caregiver survey was for parents whose child received the HPV vaccine, and the young adult survey was for young adults who received the HPV vaccine at 1 of the funded minigrant sites. These 2 surveys were offered in English and Spanish, and in electronic and paper formats. The staff survey was for any medical professional or staff member at a participating health department site enrolled in the minigrants program and was only offered electronically.

*Caregiver and young adult surveys*. From November 2023 to February 2024, survey data were collected from caregivers and patients aged 18 years or older who received at least 1 dose of the HPV vaccine series at 1 of the 4 minigrant sites. Four flyers, 2 in English and 2 in Spanish, with a QR code to the survey were disseminated. One flyer was for parents whose child received a dose of the HPV vaccine series, and the other flyer was for young adults who received a dose of the HPV vaccine series. The survey took approximately 15 to 20 minutes. The survey inquired about caregivers’ or young adults’ interactions with their health care providers, observation or access to educational materials at the health department, parents’ experiences and reasons for visiting the health department, as well as items related to vaccine reminders and uptake.

*Staff surveys*. We collected survey data from staff members who helped implement the levels and strategies chosen by their minigrant site between January and February 2024. The Emory CPCRN team sent an invitation email to the minigrant site leads, who then forwarded the email with the survey link to the rest of their team and staff. The survey asked about intervention characteristics, the implementation process, organizational traits, and early sustainability of the intervention. Sustainability capacity was measured using the Clinical Sustainability Assessment Tool (CSAT) (31). CSAT measures determinants of sustainability and comprises items in 7 domains: engaged staff and leadership, engaged stakeholders, monitoring and evaluation, organizational context and capacity, workflow integration, planning and implementation, and outcomes and effectiveness (31). Staff rated the statements about each domain using a 5-point scale with 1 being “to little or no extent” to 5 being “to a very great extent.” Higher scores indicated greater sustainability capacity. We also measured implementation outcomes related to the multilevel intervention using a modified version of Weiner’s implementation outcomes (28). This scale measures the acceptability, appropriateness, and feasibility of the intervention through 2 items. Staff rated these items on a 5-point scale of 1 being “completely disagree” to 5 being “completely agree.” Higher scores reflected greater agreement about those outcomes.

*Staff interviews*. Semi-structured interviews were conducted with staff members who assisted with implementing the multilevel intervention at their minigrant site. From February to March 2024, the Emory team conducted 5 interviews. The interview domains consisted of factors leading to the adoption of the program, intervention implementation, thoughts about intervention characteristics, factors inside and outside of their organization that helped with implementation, the implementation process, program maintenance, and technical assistance. We broadly applied the 5 CFIR domains in the items related to intervention delivery (individual and innovation characteristics) and inner and outer setting factors (30). The interviews lasted an hour and were conducted and recorded over Zoom (Zoom Communications, Inc). Interviews were transcribed verbatim for data analyses.

### Data analyses

We conducted all analyses in Excel for the surveys and used MAXQDA 24 for the qualitative analyses (32). Descriptive statistics were run, and scales (ie, implementation outcomes, CSAT) were calculated. For the qualitative analyses, we developed the codebook from the verbatim transcripts, and subsequent transcripts employed the codebook with minor changes (33). Data were analyzed by using deductive coding (eg, interview questions related to benefits, delivery, and CFIR domains) and thematic analysis with constant comparison (33), which included 1) reviewing and coding the transcripts, 2) comparing different responses to identify themes, 3) coding additional interviews, and 4) refining or adding new themes. Two coders used a finalized codebook to code all transcripts, and quotes were selected to represent major themes. We described where data converged and where interviews expanded our understanding of the survey implementation ratings of the multilevel intervention (27).

## Results

### Reach and adoption of intervention levels

All 4 minigrant sites implemented the individual level and most focused on the practice level (Table 1). One site chose to implement all 3 levels. Common individual activities that were used included educational materials, patient reminders, and patient incentives for receiving the HPV vaccine. Other outreach activities included community outreach events at school fairs or education to other partners. Provider education or lunch and learns were given at the provider level, and at the practice level, health departments posted HPV information on signage or electronic boards, implemented standing orders (ie, protocols that allow nonphysician personnel, such as nurses or medical assistants, to vaccinate without direct involvement of a physician at the time of vaccination administration), reduced structural barriers (eg, change of clinic times or workflow, navigation, methods for transportation, or language barriers), and conducted education through other clinical services (eg, influenza clinic).

**Table 1:** Characteristics of 4 Funded Health Departments and Intervention Outcomes, Minigrant Program to Promote HPV Vaccination Among Adolescents in Rural Georgia, 2023–2024

Characteristic	Health department				Sites A-D
	Site A	Site B	Site C	Site D	
Award amount and length^a^	$9,080 for 12 months	$10,000 for 12 months	$8,800 for 12 months	$10,000 for 12 months	—
Target audience age, y	11–26	11 to ≥26	9–26	13–17	—
Levels implemented	Individual and practice	Individual and practice	Individual, provider, and practice	Individual and provider	—
**Intervention activities**					
Individual	• Mailed letters to patients about vaccine (reminders) • Patient incentives • Provided information at back-to-school events	• Patient incentives (gift cards) and increase visibility on website and FaceBook • Patient reminders • Met with local colleges to promote vaccine	• Placed new flyers in building • Patient incentives for vaccine • Patient reminders	• Distributed HPV information sheet to parents • Sent out patient reminder cards with an incomplete series • Small media: gave out magnet cards to those initiating the vaccine	—
Practice	• Provided information to offsite influenza clinics about the vaccine • Reduced structural barriers (eg, transportation)	• Provided staff and public information about this program • Extended clinic hours (ie, a structural barrier)	• Added cervical cancer message to new electronic sign • Standing order	NA	—
Provider	NA	NA	Provider training	Provider training	—
**HPV vaccine outcomes^b^**					
**Total vaccinations**					
2022	36	316	125	70	547
2023	55	432	146	60	693
Percentage change	52.8	36.7	16.8	−14.3	26.7
**Total initiation**					
2022	10	117	41	23	191
2023	15	210	45	20	290
Percentage change	50.0	79.5	9.7	−13.0	51.8

Abbreviations: HPV, human papillomavirus; NA, not applicable.

a Award amount varied based on funds requested in the application.

b HPV vaccine uptake data from immunization records from 2022 (before intervention) versus 2023 to 2024 1st quarter (5 quarters). Percentage change is the difference from 2022 to 2023; positive numbers indicate more vaccinations while negative numbers indicate fewer vaccinations.

### HPV vaccination

Total HPV vaccinations increased from 547 to 693 (26.7%) from the year before the program to the implementation year (1 year and the first quarter of 2024) (Table 1). The number of vaccinations increased from 191 to 290, a 51.8% increase. Differences were found in the number of vaccinations (both initiation and completion) between sites, with 1 site having a slight decrease in overall vaccination.

### Implementation perspectives

*Caregiver and young adult surveys*. Twenty caregivers and 13 young adults responded to the follow-up survey. Caregivers and parents were mostly female (70%), aged 18 to 50 years (80%), Black (60%), non-Hispanic (94%), and single (56%). Most had less than a college degree (89%) and an income of $50,000 or less (61%). Young adults were mostly female (77%), younger than 26 years (92%), Black (46%) or White (46%), and non-Hispanic (92%). Caregivers and young adults corroborated the activities selected by the health departments (n = 20), with 80% and 77%, respectively, reporting that providers discussed the vaccine; 90% of caregivers and 83% of young adults saw or received materials in the follow-up survey. More caregivers reported that they received reminders for future vaccinations than did young adults (75% vs 46%) (Table 2A). Both groups that had their child or themselves receive the vaccine had high trust in the recommendations of the providers that they met with (parents, mean [SD] = 4.6 [0.6]; young adults, mean [SD] = 4.9 [0.3]) (Table 2B).

**Table 2A:** Parent, Caregiver, and Young Adult Observations of the Multilevel Intervention, Minigrant Program to Promote HPV Vaccination Among Young Adults in Rural Georgia, 2023–2024

Statement	% of Parents or caregivers who responded yes (n = 20)	% of Young adults who responded yes (n = 13)
Has a doctor or other health care provider at this health department ever recommended the HPV vaccine to you?	80	77
Did the doctor or other health care provider at this health department tell you that the HPV vaccine prevents cancer?	55	69
Before you received the HPV vaccine/dose, did you *see any educational materials* about HPV or the vaccine at your health department?	100	69
What type of educational material did you see?		
Pamphlet	28	36
Poster	28	NA
Flier	22	27
Before you received the HPV vaccine/dose, did you receive any educational materials about HPV or the vaccine?	90	83
Did you receive any type of reminders?	75	46

Abbreviations: HPV, human papillomavirus; NA, not applicable.

**Table 2B:** Parent, Caregiver, and Young Adult Perspectives on Educational Materials From the Multilevel Intervention, Minigrant Program to Promote HPV Vaccination Among Young Adults in Rural Georgia, 2023–2024^a^

Perspective	Parents or caregivers, % who agreed or strongly agreed	Parents or caregivers, mean (SD)	Young adults, % who agreed or strongly agreed	Young adults, mean (SD)
The material was easy to understand.	20 (100)	4.7 (0.5)	11 (100)	4.9 (0.3)
HPV and HPV related-cancer information I received from clinic staff was useful.	20 (100)	4.6 (0.5)	11 (100)	4.9 (0.3)
I trust the information about HPV and HPVrelated cancer from a doctor or other health professionals.	19 (95)	4.6 (0.6)	11 (100)	4.9 (0.3)
I feel comfortable discussing my questions or concerns about the HPV vaccine with the provider and other clinic staff.	18 (95)	4.6 (0.6)	12 (91)	4.6 (0.7)
The educational material influenced my decision to receive the HPV vaccine for my child/children.	17 (85)	4.4 (0.8)	11 (100)	4.9 (0.3)

Abbreviation: HPV, human papillomavirus.

a Respondents rated these items on a 5-point scale, with 1 being “strongly disagree” to 5 being “strongly agree.” Values for n varied because not all participants responded to every question.

*Staff implementation survey*. Five staff members in total completed the survey; all sites were represented, and 2 were from the same site. They all identified as white (80%) and female (100%). Most worked as county nurse managers (40%) or registered nurses (40%) and reported that they had been with the department for less than 5 years (60%). Overall, health department staff reported positive experiences with program implementation and perceived the program was feasible (mean = 4.4), acceptable (mean = 4.4), and appropriate (mean = 4.2). They also reported high ease of implementation (mean = 4.0) and commitment to program delivery (mean = 4.8) (Table 3).

**Table 3:** Staff Attitudes and Implementation Outcomes, Minigrant Program to Promote HPV Vaccination Among Young Adults in Rural Georgia, 2023–2024 (N = 5)

Statement^a^	Disagree, No. (%)	Neutral, No. (%)	Agree, No. (%)	Strongly, agree No. (%)	Mean (SD)
The intervention was a good match for my organization.	0	0	4 (80)	1 (20)	4.2 (0.5)
The intervention aligned with the mission of my organization.	0	0	2 (4)	3 (60)	4.6 (0.6)
The program was easy to implement at my site.	0	1 (20)	3 (6)	1 (20)	4.0 (0.7)
The evidence-based program is suitable.	0	0	4 (80)	1 (20)	4.2 (0.5)
Using this intervention is more effective than our prior practices for increasing HPV uptake.	0	0	4 (80)	1 (20)	4.2 (0.5)
People who work here are determined and motived to implement the HPV vaccination program.	0	0	1 (20)	4 (80)	4.8 (0.5)
The culture of my organization helped me implement the program.	1 (20)	0	3 (60)	1 (20)	3.6 (1.5)
We get support from colleagues outside of our practice for implementing this intervention besides Emory University.	3 (60)	0	1 (20)	1 (20)	2.6 (1.8)
Leadership is very involved in the implementation of the HPV vaccination program.	0	0	3 (60)	2 (40)	4.4 (0.6)
**Implementation outcomes^b^**					
**Intervention acceptability **					
This program meets my approval.	0	0	2 (40)	3 (60)	4.6 (0.6)
This program is appealing to me.	0	0	4 (80)	1 (20)	4.2 (0.5)
**Intervention appropriateness**					
This program is fitting.	0	0	4 (80)	1 (20)	4.2 (0.5)
This program is suitable.	0	0	4 (80)	1 (20)	4.2 (0.5)
**Intervention feasiblity**					
Program seems doable.	0	0	2 (40)	3 (60)	4.6 (0.6)
This program is easy to use.	0	1 (20)	2 (40)	2 (40)	4.2 (0.8)
Composite score	—	—	—	—	4.3 (0.6)
**Effectiveness statements^a^**					
The program is effective in increasing HPV vaccine initiation rates among the program population.	0	0	4 (80)	1 (20)	4.2 (0.5)
The program is effective in increasing HPV vaccine completion rates among the program population.	0	0	5 (100)	0	4.0 (0.0)

Abbreviations: HPV, human papillomavirus; —, not applicable.

a Staff rated these items on a 5-point scale, with 1 being “strongly disagree” to 5 being “strongly agree.” Higher scores reflected greater agreement about these outcomes.

b Staff rated these items from 1 being “completely disagree” to 5 being “completely agree,” with neutral responses being coded as “neither agree nor disagree.”

*Interviews with staff*. Five staff members (all survey participants) completed interviews; 2 participating staff members were from the same site. Most staff members were either the county nurse manager or the immunization coordinator (80%), had been at the organization less than 5 years (60%), were female (100%), and were White (80%). Across all interviews, health department staff expressed that this was the department’s first time focusing on HPV vaccine promotion efforts. They all similarly shared that the intervention strategies were straightforward to implement and found that gift card incentives were among the most effective strategies in getting people vaccinated, which validated the overall survey rating that the intervention was easy to implement. Although some strategies were more effective than others, staff recognized the importance of a multilevel approach. After the minigrants program concluded, all departments shared that they will continue promoting the HPV vaccine to their patients. For themes regarding what would help them continue to deliver the program, staff mentioned funding, particularly for the gift card as client incentives and use of the GRITS (Georgia Registry of Immunization Transactions and Services) immunization system. Workflow is a critical sustainability element and is one of the highest-rated indicators on the CSAT, and the finding that staff used patient databases aligns with this element. 

### Implementation barriers and facilitators

Related to CFIR themes, we identified facilitators and barriers to intervention delivery across the domains (Table 4). For facilitators, the staff expressed that leadership was supportive in pushing HPV vaccine promotion efforts forward and encouraged collective work across the department throughout program implementation. CFIR-related facilitators were a relative priority of the topic, leadership involvement, presence of technology infrastructure, communications about the vaccine and program, available resources (eg, minigrant funds, toolkit materials), planning for vaccination education, reminders and access to knowledge (staff training) at the inner setting and external support at the outer setting. For joint analyses, there were high ratings of the alignment of the program with their mission, leadership support in pushing HPV vaccine promotion efforts forward and encouragement of collective work for the HPV intervention delivery on the survey. 

**Table 4:** CFIR-related Facilitators and Barriers to Implementation, Minigrant Program to Promote HPV Vaccination Among Young Adults in Rural Georgia, 2023–2024

Facilitators to implementation and subcode	Theme	CFIR construct	Quotes
**Domain: inner setting**			
Prior experience	Expanding on HPV vaccination program	Mission alignment	“Not like we focused on it last year. No. Of course we’ve offered it and we’ve had pamphlets before our HPV that we gave out at different places. The way the staff promoted it and during the last year, no, they went above board, did more.” Participant 2
Resources	Funds for the program and incentives	Available resources	“Well, this one, this one worked mainly worked a lot better. It was just because we had so many resources. I was trying to do that basically what me and a clerk would try to work that trying to get that together with very limited resources. This one has been very — a lot better getting people to come in. I didn’t have incentives to get patients to come back. It didn’t — I think just sending out reminders to people who start ’em a lot of times will help because six months is a long time, and they forget.” Participant 3 “All of the resources and then the reminders you all gave us, the magnets, all of that, the postcards, the incentives, it worked really well to help us get these people in to get their vaccine or at least get ’em to start it.” Participant 3
HPV vaccination as a priority	Promoting the vaccine is a priority for everyone at the department	Relative priority	“I do. I do, especially for the — for our — ’cause we promote it even in our family planning program too. We try to get those patients to take family planning, STD, whatever program they're coming in. We do try to encourage ‘em to take it not just when they're coming in for school shots or just coming here for shots. We do it in all programs. I do think it's a priority.” Participant 3 “Yes, it is. I think that even though we will, after this month, stop giving out the incentives. I think that they're still — it’s just kind of ingrained in them to promote it. I believe they’ll continue to do that. Well, they already realize how important it was, but it is just, I guess with all the education that’s gone on and — I mean, it’s just been in the forefront of their mind and it’s sort of like a habit — it’s something that they’ve learned, I think they’ll continue to do it.” Participant 2
Communications/social media	Using Facebook, Twitter, and Tik Tok to communicate with patients	Information technology infrastructure	“Facebook was effective, because people would call.” Participant 5 “I know we advertised on our — we’ve got like a billboard electronic sign, and we did that for the majority of the year. I think we still actually have something out there now.” Participant 3
Patient communications	Communication methods to patients	Information technology infrastructure	“Well, staff one thing ’cause we had extra staff on the clerks. We had one in particular. . . . She would actually be the one that we would give her the list. She would call and try to do all the calls to the patient before we actually sent out the — when we were doing the letters.” “’Cause it costs money, and then it they come back, then you’ve wasted that money.” Participant 3
**Domain: process of implementation**			
Facilitators to implementation	Education for staff and internal support, as well as intervention materials	Tailoring strategies and engaging	“I think just everybody here being on board with implementing the program. Not letting their own personal opinion affect implementing the program.” Participant 5 “I think some of the — as far as getting the materials that we got out made the delivery easier as far as I know any time doing the message outside may get people here easier, but some — a lot of our materials that we have, if it talks about cancer or stuff like that, I does make it easier to get patients to be receptive of anything, any kind of intervention we’re doing as far as with HPV and getting them to actually respond or do that. Probably a lot of the actual materials that — even — ’cause even like on the magnets, we had the information on those and the cards. I think that, giving people that message helps better than giving I would say money.” Participant 3 “They were excited about the incentive, the gift cards. I really think that they liked the magnets ’cause we don't have any other — we don't typically give out any kind of reminder like that. A lot of the kids liked those.” Participant 5
Leadership involvement	Support and involvement from supervisors and board members along with team meetings with leadership	Individual domain-high level leaders or executing	“. . . One of our Board of Health members is a doctor that works at the Primary Health Care. It’s a federally-qualified-health center. He’s on our board, and he actually was an advocate for this whole HPV grant, so we discussed it in our Board of Health thing, and he took it back to the Primary Health Care side, and even to the facilities that are in Albany, he was tellin’ them, ‘You know public health [X] County and [Y] County have grants, and we need to be tellin’ our patients, so they need to go and and get.’ He was very specific, ‘I want to know what ages you-all are doin’ and are you offerin’ to people over 26 because we have a big clientele.’ He was very in on that too.” Participant 4 “I've had no problems with any leadership. I think that they’ve supported this fully. I’ve tried to support it fully, so I think it's been very positive.” Participant 1
Changes in implementation	Planning for patient education vaccine reminders appointments	Planning	Here, it starts from the time the patient comes in the door, whenever they get to our clerk’s desk. The clerk tells them, “These are the shots that are required for your child for school. These are the shots that are recommended.” Then they provide them with the VIS statements regarding all of the vaccines. Typically, the parents are like, “Oh, I didn't know.” Even for — we have college-aged students that come in, and it’s the same conversation with them like, “These are the vaccines we know that will typically be required by a college, but these are the vaccines that are also recommended.” Just presenting it that way. Participant 1 Previously, we weren't doing a lot of that, scheduling the next appointments. People would just call. We would give ’em a day, and they would just call and schedule their appointment. But we went ahead and started making them appointments now for their next vaccine. Participant 4
Sustaining the Program	Availability of immunization records funds		Well, we’re definitely keepin’ the strategy of the clerk printin’ the GRITS record for everybody that comes in. . . . That was something that was implemented a long time ago, and I think it kinda fell to the way side a little bit, but definitely, that’s put back in our forefront of our mind again. I think that was probably one of the best ones, and definitely, on the recall. I’ll probably utilize that several times. Participant 2 In the future, as far as — I think we could do — I mean ’cause you know we have to input anyway for our [educational/reminder] cards and our letters and stuff, so that shouldn't be a problem. Like I said, unless we get funding somewhere, we're probably won't continue incentives, not that much of an incentive. The majority of everything else we could still do. I could still go to the school every year and do that. I have to go anyway, so I might as well. Participant 3 It'd be nice to have some funds, but if you don’t, then I think we just keep doing what we did. It doesn’t cost money, which is the education piece, the talking to other providers, word of mouth, staying close to the school nurse to help her to encourage that also. Participant 4
**Domain: outer setting**			
External support	Partnerships and connections	Relational connections	“Well, I know at that — I think it was mentioned with the school system about them reminding parents that the children were eligible for the HPV vaccine also, in addition to what was required for school. I want to say there was somethin’ that went — I can’t speak specifically, but with Albany State and an initiative with them to increase HPV. I don’t know if they — they may have taken the vaccines out to the school. I can’t remember specifically. I feel like there was somethin’ that happened with them to increase.” Participant 5 “I think the hospital because they were real supportive with it, and the school system, I mean.” Participant 1
External Communication	Societal pressure	Tension for change	“They would go tell their friend, hey, go out there and get that shot. It didn’t hurt at all, and you get a gift card for doing it. Plus it helps prevent cancer, so yeah.” Participant 1
**Barriers to implementation**			
**Domain: inner setting**			
Communications and Engagement	Communicating about HPV vaccine	Implementation-engaging innovation recipients	“Changed it up like what we would say some of it would — sometimes it would be cancer prevention, ask us about our vaccine that kind of advertisement. We also use flyers. I did take — contact with some of the local schools. Then the providers, we did the lunch and learns with the providers.” Participant 3
	Outdated EMR system to verify vaccination	Information technology infrastructure	“One of my biggest pet peeves is going into GRITS and seein’ that there’s 5 or 6 different responsible parties and different addresses and different phone numbers, and that has not been cleaned up.” Participant 4
	Communication methods to patients	Information technology infrastructure	“We’re not allowed to do email either.” Participant 5
Enhancing infrastructure and new resources	First time implementing HPV vaccination program	Structural characteristics and available resources	“Well, this one, this one worked mainly worked a lot better. It was just because we had so many resources. I was trying to do that basically what me and a clerk would try to work that trying to get that together with very limited resources. This one has been very — a lot better getting people to come in. I didn't have incentives to get patients to come back. It didn’t — I think just sending out reminders to people who start ’em a lot of times will help because six months is a long time, and they forget.” Participant 3
**Domain: outer setting**			
People who lack knowledge or are hesitant	Vaccine hesitancy	Local attitudes	“We had a few people that even with the incentives, they weren’t gonna take the shot regardless, but honestly, that was just a — it really is not — I noticed ’cause I’ve been doing this so long.” Participant 3 “Definitely the education piece because a lot of people, they’ve heard of the Gardasil shot or the HPV shot, but they don’t really know what it is or if they need it. I think that that's just a really big piece of it, just the education piece.” Participant 1
Transportation	Lack of transportation infrastructure	Local conditions	“The only barrier I guess I see is, sometimes transportation can be a barrier because we don’t have like public transportation here. Some of our patients live 15, 20 miles away. You might get ’em up here once, and then for them to get back is very difficult. I think that’s one of our major barriers.” Participant 1
Outreach to healthcare providers	Meeting and educating providers	Partnerships and connections	“I think the most difficult one was that [staff member] wanted to do was the getting in there with the providers, meeting with them. To me that seemed like the — just timewise, you can’t — or staffing issues, that was probably the most difficult, trying to meet with someone face-to-face all the time, especially this day and age ’cause most people can't do that anymore hardly ever.” Participant 3 “Time, having the time to do stuff with the providers was probably the biggest barrier with that intervention.” Participant 3

Abbreviations: CFIR, Consolidated Framework for Implementation Science; EHR, electronic health record; GRITS, Georgia Registry of Immunization Transactions and Services; HPV, human papillomavirus.

Barriers centered around CFIR inner setting domains and were mostly around communication and structural characteristics (eg, technology/electronic health record [HER]) (Table 4). Challenges related to outer setting domains included willingness of people to use the vaccine or hesitancy to use it, transportation issues for accessing health care (ie, local attitudes and conditions), and reaching other providers (ie, partnerships) about vaccine promotion.

### Program sustainability capacity

The mean overall program sustainability capacity score was 4.2 (SD, 0.6) with highest scores for domains of monitoring and evaluation (mean [SD] = 4.5 [0.5]), outcomes and effectiveness (mean [SD] = 4.4 [0.5]), workflow integration (mean [SD] = 4.2 [0.5]), and implementation and training (mean [SD] = 4.2 [0.8]) (Table 5). Most staff agreed to a great extent that they would continue to offer the program (mean [SD] = 3.8 [0.8]). Related to training and technical assistance during the minigrants period, staff agreed that the training and technical assistance offered was helpful (mean [SD] = 4.6 [0.6]) and that they plan to continue to use the various web-based resources and toolkit (mean [SD] = 4.6 [0.6]) (data not shown).

**Table 5:** Sustainability Capacity of a Minigrant Program to Promote HPV Vaccination Among Young Adults in Rural Georgia, 2023–2024 (N = 5)^a^

Domain and item	Mean (SD)
**Engaged staff and leadership**	
The program engages leadership and staff throughout the process.	4.0 (0.7)
Clinical champions of the program are recognized and respected.	3.6 (0.9)
The program has engaged, ongoing champions.	3.8 (0.5)
The program has a leadership team made of multiprofessional partnerships.	4.0 (0.7)
The program has team-based collaboration and infrastructure.	4.0 (0)
Engaged staff and leadership composite score	3.9 (0.6)
**Engaged stakeholders**	
The program engages the patient and family members as stakeholders.	4.0 (0)
There is respect for all stakeholders involved in the program.	4.2 (0.5)
The program is valued by a diverse set of stakeholders.	4.0 (0.7)
The program engages other medical teams and community partnerships as appropriate.	4.0 (0)
The program team has the ability to respond to stakeholder feedback about the program.	3.8 (0.5)
Engaged stakeholders composite score	4.0 (0.4)
**Organizational context and capacity**	
Organizational systems are in place to support the various program needs.	3.8 (0.5)
The program fits in well with the culture of the team.	4.0 (0)
The program has feasible and sufficient resources (eg, time, space, funding) to achieve its goals.	4.4 (0.6)
The program has adequate staff to achieve its goals.	4.0 (1.0)
The program is well integrated into the operations of the organization.	4.0 (0)
Organizational readiness composite score	4.0 (0.5)
**Workflow integration**	
The program is built into the clinical workflow.	3.8 (1.1)
The program is easy for clinicians to use.	4.2 (0.5)
The program integrates well with established clinical practices.	4.2 (0.5)
The program aligns well with other clinical systems (eg, EMR).	4.2 (0.5)
The program is designed to be used consistently.	4.4 (0.6)
Workflow composite score	4.2 (0.6)
**Planning and implementation**	
The program clearly outlines roles and responsibilities for all staff.	4.2 (0.8)
The reason for the program is clearly communicated to and understood by all staff.	4.2 (0.8)
Staff receive ongoing coaching, feedback, and training.	4.2 (0.8)
Program implementation is guided by feedback from stakeholders.	4.0 (0.7)
The program has ongoing education across professions.	4.2 (0.8)
Implementation composite score	4.2 (0.8)
**Monitoring and evaluation**	
The program has measurable process components, outcomes, and metrics.	4.4 (0.6)
Evaluation and monitoring of the program are reviewed on a consistent basis.	4.6 (0.6)
The program has clear documentation to guide process and outcome evaluation.	4.6 (0.6)
Program monitoring, evaluation, and outcomes data are routinely reported to the clinical care team.	4.4 (0.6)
The program process components, outcomes, and metrics are easily assessed and audited.	4.4 (0.6)
Monitoring composite score	4.5 (0.5)
**Outcomes and effectiveness**	
The program has evidence of beneficial outcomes.	4.4 (0.6)
The program is associated with improvement in patient outcomes that are clinically meaningful.	4.4 (0.6)
The program is clearly linked to positive health or clinical outcomes.	4.4 (0.6)
The program is cost-effective.	4.4 (0.6)
The program has clear advantages over alternatives.	4.6 (0.6)
Outcomes composite score	4.4 (0.5)
Total CSAT score	4.2 (0.6)

Abbreviations: CSAT, Clinical Sustainability Assessment Tool; EMR, electronic medical record.

a Staff rated the statements about each domain using a 5-point scale with 1 being “to little or no extent” to 5 being “to a very great extent.” Higher scores indicate greater sustainability capacity.

## Implications for Public Health

Overall, we found that this pilot minigrants program of multilevel interventions to promote HPV vaccination had a positive impact. There was a 27% increase in HPV vaccinations overall from the year before the year of the implementation of the multilevel intervention, with a 52% increase in HPV vaccine initiation across sites. Most of the health departments experienced increases in both vaccine initiation and overall total vaccinations. Two of the sites with the largest increases were focused on evidence-based strategies at the individual and practice levels, which may mean that future interventions to promote HPV vaccine uptake do not have to focus on all 3 levels. This study contributes to the literature of the impact of minigrants for cancer prevention and control in local communities (24,34). Other multilevel interventions to promote HPV vaccination have found improvements in vaccination (35–37).

Overall staff had fairly positive experiences with the multilevel intervention. Most agreed that it was more effective than their prior practices and that it was easy to implement. They also reported that the program was highly acceptable, slightly feasible, and appropriate for implementation outcomes. From the interviews with health department staff, many CFIR-related facilitators existed for the implementation of the multilevel interventions including leadership support, resources, staff training about the HPV vaccine, advance implementation planning, and partnerships. Other research showed similar results with use of EHR systems for reminders and monitoring, training and education, concrete tools and resources, strong leadership support, provider recommendation, and provider champions as facilitators to implementing HPV vaccination interventions (38,39). Barriers to the multilevel interventions included limited resources and staff, communication and structural characteristics (eg, technology/EHR data at the individual site) at the inner setting, and local attitudes about vaccination, outreach to other providers to increase vaccination and transportation at the outer setting. These results validate other research that cite infrastructure (eg, EHRs), competing priorities, available resources (ie, funding), staff buy-in, training needs, provider characteristics such as knowledge about vaccines, and confidence in relaying information. For the outer setting, elements include patient misinformation about vaccines and vaccine stigma (local attitudes), cultural and language barriers, and lack of public media about vaccination (38–40). Critical incidents such as the COVID-19 pandemic and HPV vaccine supply chain problems are other recent challenges to HPV vaccination programming (40). Given the lower HPV vaccination rates, it is critical to understand implementation challenges in rural areas as public health and researchers promote HPV vaccination in rural communities. Factors external to the organization — including tailored health education and campaigns, addressing health care access, and vaccine hesitancy, in addition to enhancing education among partners in communities — need to be explored to increase rural vaccination rates.

Maintenance of these interventions was perceived by staff to be contingent on evaluation results, monitoring of process, and integration of the intervention into each department’s workflow and training of staff. This program offers proof of concept for implementation strategies of funding (minigrants), a learning collaborative and technical assistance; additional investments may be needed to support the delivery of these evidence-based multilevel grants. Others have called for additional research on the long-term implementation and sustainability of HPV vaccine programs (41). Additionally, 1 funded site did not have increases in total vaccination. This could be that we were working with small, rural health departments in southwest Georgia. These departments may have additional barriers to the conduct of multilevel interventions such as limited personnel, vaccine hesitancy, or other resources despite receipt of minigrant funds and technical assistance. Future capacity-building projects related to promotion of HPV vaccine uptake can assess if there are additional supports needed for diverse types of sites.

Strengths of this implementation evaluation were the multilevel interventions, mixed-methods data collection, and application of implementation science theories. However, our study has several limitations. It was based on the minigrants work of only 4 public health departments, so the findings may not be generalizable. We examined data on HPV vaccinations only for the full year and first quarter (5 quarters) of implementation of the intervention through general data-sharing agreements. Extending the timeframe of the evaluation could offer insights into the longer-term effects of the program. Future evaluations or quality improvement initiatives should assess long-term effects of these implementation strategies. In addition, completion of the parent and caregiver and young adult surveys varied by site (range, 4 to 20). Even so, all sites had survey responses, which validated the multilevel intervention components. Social desirability (response bias) and recall biases may be evident on the parent and caregiver, young adult, and health department surveys. However, we found that the intervention components reported by health departments were corroborated by parents’ and young adults’ survey data and by the staff workplans. Finally for vaccine outcomes, we were able to report on total HPV vaccine shots and initiation and not on completion, relying on the health department vaccine records. In addition, we cannot verify the data quality of the medical records in terms of missingness or accuracy since they were provided as part of the evaluation. Vaccine completion is more difficult to assess due to the study period limits and not having identifiable patient-specific data on the evaluation; data on ongoing vaccine completion for sites is required for further data analyses. Future implementation of the program could extend the data collection timeframe and assess vaccine completion outcomes.

Multilevel intervention strategies can successfully increase HPV vaccination rates in rural settings with implementation supports to address cancer prevalence in rural areas.

Our minigrants program to support implementation of multilevel HPV interventions was successful in increasing HPV vaccination across health department sites. The evaluation showed that the interventions were easy to implement at the health departments and had high acceptability and feasibility. Future research can expand on minigrants and these implementation strategies and examine ways to sustain these programs to increase HPV vaccination in rural communities to reduce cancer health disparities.
